# Nanocomposites of Polyhydroxyurethane with POSS Microdomains: Synthesis via Non-Isocyanate Approach, Morphologies and Reprocessing Properties

**DOI:** 10.3390/polym14071331

**Published:** 2022-03-25

**Authors:** Weiming Liu, Guohua Hang, Honggang Mei, Lei Li, Sixun Zheng

**Affiliations:** Department of Polymer Science and Engineering, The State Key Laboratory of Metal Matrix Composites, Shanghai Jiao Tong University, Shanghai 200240, China; weiming.liu@sjtu.edu.cn (W.L.); hollmes@sjtu.edu.cn (G.H.); mhg2018@sjtu.edu.cn (H.M.)

**Keywords:** five-membered cyclic carbonate, POSS, polyhydroxyurethane, nanocomposites, reprocessing properties

## Abstract

In this contribution, we reported the synthesis of a novel trifunctional POSS cyclic carbonate [POSS-3(5CC)]. With a difunctional five-member cyclic carbonate and a trifunctional polyetheramine as the precursor, the nanocomposites of polyhydroxyurethane (PHU) with POSS were synthesized. Transmission electron microscopy (TEM) showed that the nanocomposites of PHUs with POSS were microphase-separated; the spherical POSS microdomains via POSS-POSS interactions were generated with the size of 20~40 nm in diameter. After the introduction of POSS microdomains, the nanocomposites displayed improved thermal and mechanical properties. More importantly, the nanocomposites still displayed the reprocessing properties of vitrimers.

## 1. Introduction

Polyurethanes (PUs) are a class of important polymers, which have wide applications in various fields such as coatings, adhesives, foams, and textiles [1,2]. Despite excellent properties, the application of PUs is subject to a big concern since one of the precursors of PUs (viz. diisocyanates) as well as the starting materials of diisocyanate (viz. phosgene) are harmful to human health and the environment. It is long a pursuit to synthesize PUs via isocyanate-free routes [3]. The polyaddition of polyfunctional cyclic carbonates with amines affords a class of new polymers, i.e., polyhydroxyurethane (PHU) which has a structure similar to PU [4,5,6,7,8,9,10,11]. Compared with the traditional PUs, there are additional side hydroxyl groups adjacent to the carbamate groups along with the main chain of PHU. Owing to this structural feature, PHUs displayed some new properties. On the one hand, the dynamic reversible transesterification reaction of hydroxyl and carbamate groups would occur at elevated temperatures, which endows PHU networks with reprocessing properties. On the other hand, the side hydroxyl groups provide the possibility further to functionalize PHU to obtain new and functional properties.

In the past years, there has been ample literature to report the synthesis and correlation of structure with properties of PHUs [12,13,14,15,16,17,18,19,20,21,22,23,24,25,26,27,28,29,30]. For instance, Endo et al. [31,32,33,34] have reported the synthetic approaches of five- and six-membered cyclic carbonates, which can be used as the monomers of PHUs. Hillmyer et al. [35] investigated the reprocessing properties of PHUs which resulted from six-membered cyclic carbonates and amines. It was demonstrated that the high recovery of mechanical properties was attained after the PHUs were reprocessed. More recently, Torkelson et al. [36] reported that in the presence of catalyst [viz. 4-(dimethylamino)pyridine (DMAP)], PHU thermosets from five-membered cyclic carbonate and amines also displayed excellent reprocessing properties. The reprocessing properties are attributable to both transcarbamoylation exchange reaction and reversible cyclic carbonate aminolysis, depending on the use of different cyclic carbonate monomers.

The incorporation of inorganics in organic polymers is an important technique to develop materials with excellent comprehensive properties. Recently, organic-inorganic nanocomposites of PHU with various inorganic nanoparticles have been also reported; the nanocomposites possess enhanced thermal, mechanical properties, and other specific properties [37,38,39,40]. For instance, Zheng et al. [37] reported PHU/carbon nanotubes (CNTs) nanocomposites. Notably, the incorporation of CNTs significantly improved the mechanical and dielectric properties of PHU. Torkelson et al. [38] reported PHU/silica nanocomposites by incorporating the surface-modified silica nanoparticles into PHU thermosets. It was found that in the process of PHU network rearrangement, the interface interaction between the silica nanoparticles and PHU matrix is essential to affect the mechanical and reprocessing properties of the materials. More recently, Zheng et al. [39] synthesized the surface modification of Fe_3_O_4_ nanoparticles with five-membered cyclic carbonates. The surface-modified nanoparticles were readily incorporated into a PHU of erythritol-based five-membered cyclic carbonate with polyetheramine and the nanocomposites were successfully obtained. It was found that the nanocomposites exhibited excellent reprocessing and laser-induced shape memory properties.

Polyhedral oligomeric silsesquioxanes (POSS) are a class of important organic-inorganic building blocks. POSS molecules have cage-like nanostructures; their corner silicon atoms were bonded with organic substituents, one or more of which is reactive or polymerizable. With these functional groups, POSS can be incorporated into organic polymers in the form of pendent groups or main chain structural units. Over the past years, mono-, di-, tetra- and octa-functional POSS macromers have been used to form the organic-inorganic nanocomposites with a variety of polymers [41,42,43,44,45,46,47,48,49,50]. Recently, investigators had also explored the synthesis of PHU-POSS nanocomposites. For instance, Mülhaupt et al. [51] synthesized PHU-POSS nanocomposites by using an octa-functional POSS five-membered cyclic carbonate. It was found that the incorporation of POSS significantly enhanced Young’s modulus and tensile strength of nanocomposites. Zheng et al. [52] reported that the nanocomposites of PHU with POSS with a difunctional POSS six-membered cyclic carbonate. Notably, the PHU-POSS nanocomposites exhibited excellent reprocessing and shape memory properties as well as improved thermomechanical properties. Compared to the nanocomposites of POSS with other polymers, nonetheless, the nanocomposites PHU with POSS remain largely unexplored.

In this contribution, we first reported the synthesis of a novel tri-functional POSS five-membered cyclic carbonate [denoted POSS-3(5CC)]. The POSS macromer had an incompletely condensed and open cage-like nanostructure, which was in marked contrast to the completely condensed POSS cages [51,52]. This trifunctional POSS was introduced into PHU resulting from a diglycidyl ether of bisphenol A (DGEBA)-based difunctional five-membered cyclic carbonate and a trifunctional polyetheramine, to obtain the nanocomposites. The purpose of this work is to examine the formation of POSS microdomains via POSS-POSS interactions while an open and incompletely condensed POSS cage was used, which is in marked contrast to the use of completely condensed and close POSS cages [51,52]. The morphologies of the organic-inorganic nanocomposites were investigated by means of transmission electron microscopy (TEM). The thermal and mechanical properties were investigated by means of dynamic mechanical thermal analysis (DMTA) and tensile mechanical tests. In the meantime, the impact of POSS introduction on the reprocessing properties of PHU was examined.

## 2. Experimental

### 2.1. Materials

Phenyltrimethoxysilane was obtained from Zhejiang Chemical Technology, China. Tetra-*n*-butylammonium iodide (TBAI), 4-dimethylaminopyridine (DMAP), 1,3-bis(hexafluoro-2-hydroxyisopropyl) benzene (BHFB), chlorodimethylsilane, allyl glycidyl ether, and sodium hydroxide were purchased from Adamas Co, Shanghai, China. Carbon dioxide (CO_2_) was supplied by Air Liquid (Shanghai) Co., Shanghai, China. Trifunctional polyetheramine under the trade name of Jeffamine-T403 was purchased from Sigma Co, Shanghai China and it had a quoted molecular weight of *M_n_* = 443 Da. Diglycidyl ether of bisphenol A (DGEBA)-based five-membered cyclic carbonate (denoted DE5CC) was synthesized following the approach of literature [53]. Organic solvents such as tetrahydrofuran (THF), methanol, toluene, and dichloromethane were supplied from Admas, Co., Shanghai, China.

### 2.2. Synthesis of POSS-3H

First, phenyltrimethoxysilane (45.540 g, 0.23 mol), sodium hydroxide (0.960 g, 0.024 mol), deionized water (5.260 g, 0.29 mol), and THF (250 mL) were added to a 500 mL flask. The reaction was carried out at 70 °C for 4 h and room temperature for 15 h. After the reaction, the solvent was removed by a rotary evaporator. The product, i.e., heptaphenyltricycloheptasiloxane trisodium silanolate [54] was obtained after further drying *in* *vacuo* at 40 °C for 24 h.

Second, heptaphenyltricycloheptasiloxane trisodium silanolate was dissolved in THF (300 mL) and then dimethylchlorolsilane (27.790 g, 0.294 mol) was added at 0 °C. The reaction was performed at room temperature for 24 h. After the reaction, the solids were filtered out and the solvent was removed via rotary evaporation. The concentrated solution was precipitated in methanol (300 mL) and the white powders were obtained. After dying in a vacuum oven at 40 °C, POSS-trihydro (POSS-3H) was obtained with a yield of 76.3%. ^1^H NMR (CDCl_3_, ppm): 0.47(*s*, 18H, -Si(C***H***_3_)_2_H), 5.01 (*s*, 3H, -Si(CH_3_)_2_***H***), 7.13~7.76 (*m*, 35H, protons of aromatic ring). ^29^Si NMR (CDCl_3_, ppm): −2.24 (*s*, ***Si***CH_3_), −76.62, −76.97, −77.61 (*s*, -O-***Si***-O).

### 2.3. Synthesis of POSS-3epoxide (POSS-3EP)

POSS-3H (10.000 g, 9.05 mmol) and allyl glycidyl ether (6.160 g, 54.00 mmol) were dissolved in toluene (50 mL). The system was purged with highly pure nitrogen for 30 min, and then Karstedt catalyst (300 uL) was injected into the flask. The reaction was performed at 90 °C for 36 h. Cooled to room temperature, the solvent was removed via rotary evaporation, and the product was obtained through extraction with dichloromethane. After removing the solvent, POSS-3epoxide (denoted POSS-3EP) was obtained and the yield was 83.0%. ^1^H NMR (CDCl_3_, ppm): 0.36 (*s*, 18H, -Si(C***H***_3_)_2_), 0.75 (*m*, 6H, -Si(CH_3_)_2_C***H***_2_CH_2_), 1.72 (*s*, 6H, -OCHOC***H***_2_), 2.58, 2.42 (*s*, 6H, -OCHOC***H***_2_), 3.01 (*s*, 3H, -C***H***OCH_2_), 3.23 (*t*, 6H, -C***H***_2_OCH_2_CH), 3.45, 3.55 (*s*, 6H, -OC***H***_2_CH), 7.13~7.76 (*m*, 35H, protons of aromatic ring).

### 2.4. Synthesis of POSS-Tri(Five-Membered Cyclic Carbonate) [POSS-3(5CC)]

POSS-3EP (13.000 g, 8.98 mmol), tetra-*n*-butylammonium iodide (TBAI) (0.500 g, 1.35 mmol), 1,3-bis(hexafluoro-2-hydroxyisopropyl) benzene (BHFB) (0.450 g, 1.10 mmol) and anhydrous toluene (15 mL) were placed in a autoclave. CO_2_ was introduced at room temperature until the pressure was increased to 6.0 MPa. The reaction mixture was stirred and heated at 80 °C for 48 h. After cooling to room temperature, the gas was released slowly. The solvent was removed, and the product was obtained through extraction with dichloromethane. After drying, POSS-3(5CC) was obtained as a yellow viscous liquid and the yield was 78.0%. ^1^H NMR (CDCl_3_, ppm): 0.36 (*s*, 18H, -Si(C***H***_3_)_2_), 0.72 (*m*, 6H, -Si(CH_3_)_2_C***H***_2_CH_2_), 1.68 (*s*, 6H, -OCHOC***H***_2_), 3.34~3.56 (*m*, 6H, -C***H***_2_OCH_2_CH), 3.45~3.59 (m, 6H, -OC***H***_2_CH), 4.22~4.42 (*m*, 6H, -CHC***H***_2_OCO), 4.72 (*s*, 3H, -CH_2_C***H***O), 7.13~7.76 (*m*, 35H, protons of aromatic ring).

### 2.5. Preparation of Nanocomposites

Typically, DE5CC (3.490 g, 7.27 mmol), POSS-3(5CC) (0.300 g, 0.19 mmol), T403 (2.210 g, 5.02 mmol) and DMAP (0.180 g, 1.51 mmol) were dispersed in THF (8 mL). The mixture was refluxed for 6 h and then poured into a mold and the reaction was performed for an additional 48 h. The solvent was removed completely under reduced pressure at 60 °C for 48 h. The mass fraction of POSS-3(5CC) in the nanocomposites was controlled to be 5, 10, 15, and 20 wt%, respectively.

### 2.6. Measurements and Techniques

The ^1^H and ^29^Si nuclear magnetic resonance (NMR) spectra were obtained on a Varian Mercury Plus 500 MHz NMR spectrometer at 25 °C. Deuterium chloroform was used as the solvent. Fourier transform infrared (FTIR) measurements were conducted on a Perkin–Elmer Paragon 1000 Fourier transform spectrometer at room temperature. Thermogravimetric analysis (TGA) was performed on a TA Q-5000 apparatus. The TGA measurements were performed out under a continuous flow of air gas from 50 to 800 °C at the heating rate of 20 °C × min^−1^. Transmission electron microscopy (TEM) was performed on a JEOL JEM-2010 high-resolution transmission electron microscope at an acceleration voltage of 120 kV. The PHU-POSS nanocomposites were crushed in liquid nitrogen and the powders were dispersed in ethanol. The suspension was then dropped on 200 mesh copper grids. After removing the solvent, the morphologies were observed. Dynamic mechanical thermal analysis (DMTA) was performed on a TA Q-800 instrument in a tensile mode. The measurements were performed in the temperature range of −20 to 150 °C at a rate of 3 °C × min^−1^. The rheological measurements were performed on a DHR-2 rheometer. The strain amplitude sweeps were performed at 80 °C in the strain range of 0.01 to 100% a constant angular frequency of 1.0 rad × s^−1^. The frequency sweeps were performed at 80 °C in the angular frequency range of 0.01 to 500 rad × s^−1^. The tensile tests were performed with a WDW-2 electron universal testing machine. The experiments were carried out with an elongation rate of 50 mm × min^−1^ at room temperature. Stress relaxation tests were performed with a TA Q800 dynamic mechanical thermal analyzer. The measurements were performed with a strain of 10% at different temperatures.

## 3. Results and Discussion

### 3.1. Synthesis of POSS-Tri(Five-Membered Cyclic Carbonate)

**Scheme 1:**
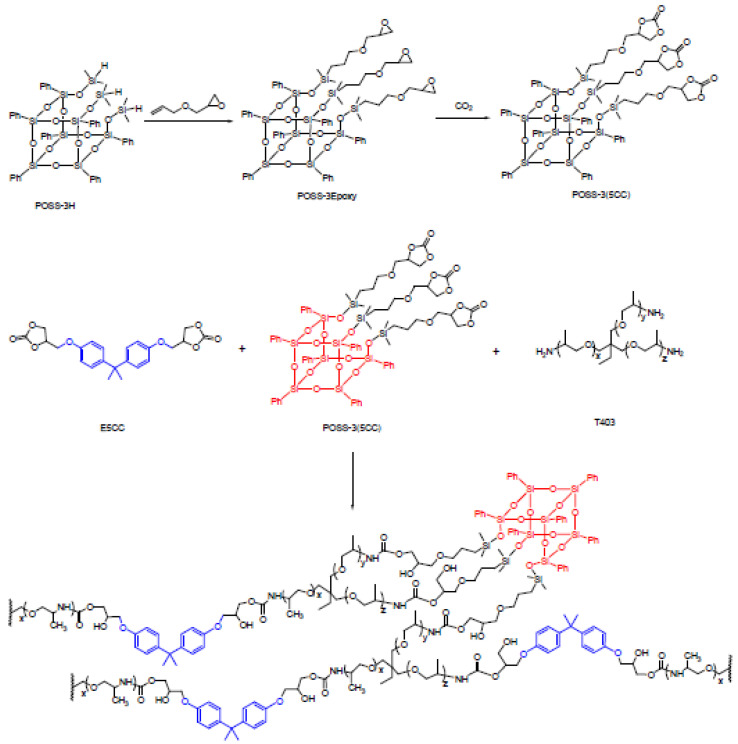
Synthesis of PHU-POSS nanocomposites.

**Figure 1:**
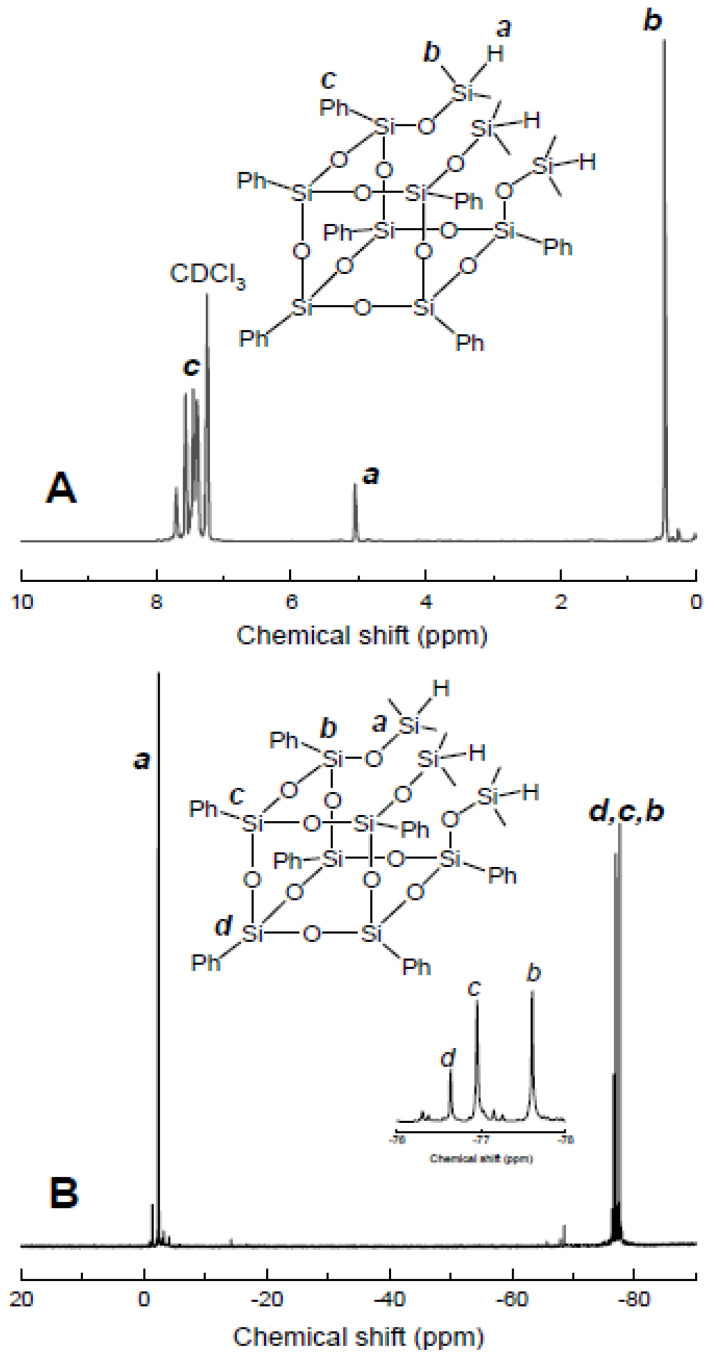
Structural characterization of POSS-3H: (**A**) ^1^H and (**B**) ^29^Si NMR spectra.

**Figure 2:**
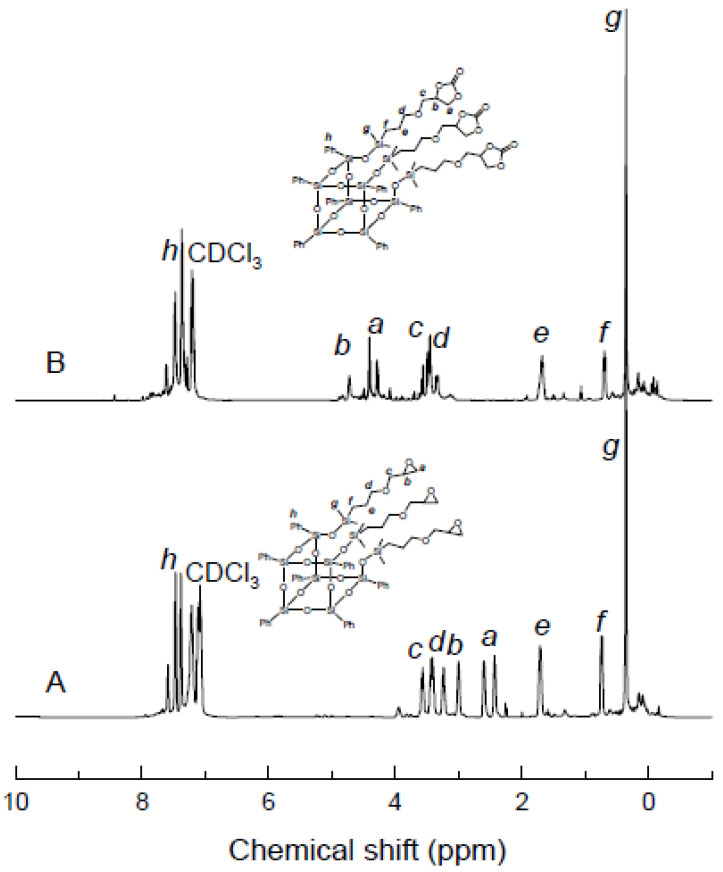
^1^H NMR spectra: (**A**) POSS-3epoxide; (**B**) POSS-3(5CC).

The route of synthesis for trifunctional POSS five-membered cyclic carbonate [denoted POSS-3(5CC)] is shown in Scheme 1. First, a POSS-trihydro macromer (denoted POSS-3H) was synthesized via the silylation reaction of heptaphenyltricycloheptasiloxane trisodium silanolate with trichlorosilane. Second, the hydrosilylation reaction of POSS-3H with allyl glycidyl ether was performed to afford POSS-3epoxide (denoted POSS-3EP) in the presence of a Karstedt catalyst. Finally, POSS-3EP was allowed to react with carbon dioxide to obtain POSS tri(five-membered cyclic carbonate [denoted POSS-3(5CC)]. The purpose to synthesize POSS-3EP is to utilize the reaction of epoxide compounds with carbon dioxide (CO_2_) to obtain five-membered cyclic carbonates, which is in marked contrast to the synthesis of POSS six-membered cyclic carbonate [52]. The ^1^H NMR and ^29^Si NMR spectra of POSS-3H are shown in Figure 1. In the ^1^H NMR spectrum (A), the signals of resonance at 0.47, 5.01, and 7.13~7.76 ppm were assignable to the protons of hydrogen atoms, the methyl groups connected with silicon atoms, and the phenyl groups, respectively. In the ^29^Si NMR spectrum (B), four different Si signals of resonance at −2.24, −76.62, −76.97, and −77.61 ppm were detected as indicated in this figure. These signals of resonance are assignable to the silicon atoms connected with methyl groups and phenyl groups, respectively. The ^1^H NMR and ^29^Si NMR results demonstrated that POSS-3H was successfully synthesized. The hydrosilylation reaction of POSS-3H with allyl glycidyl ether was carried out to obtain POSS-3epoxide (denoted POSS-3EP). As shown in Figure 2A, the signals of resonance at 5.01 ppm from the Si-H bond fully disappeared after hydrosilylation. In the meantime, the signals of resonance at 2.42, 2.58, and 3.01 ppm were detected, assignable to the protons of methylene and methane groups of terminal epoxide groups for POSS-3EP. The ^1^H NMR spectroscopy indicates that POSS-3EP was successfully obtained. The as-obtained POSS-3EP was employed to react with carbon dioxide (CO_2_) to obtain POSS-tri(five-membered cyclic carbonate) [denoted POSS-3(5CC)]. Compared to the ^1^H NMR spectrum of POSS-3EP, the signals of resonance at 2.42, 2.58 and 3.01 ppm fully shifted to 4.22, 4.42, and 4.72 ppm as shown in Figure 2B. All these peaks of resonance are characteristic of the protons from cyclic carbonate groups. The ^1^H NMR results indicate that POSS-3(5CC) was successfully synthesized.

### 3.2. Nanocomposites of PHU with POSS

**Figure 3:**
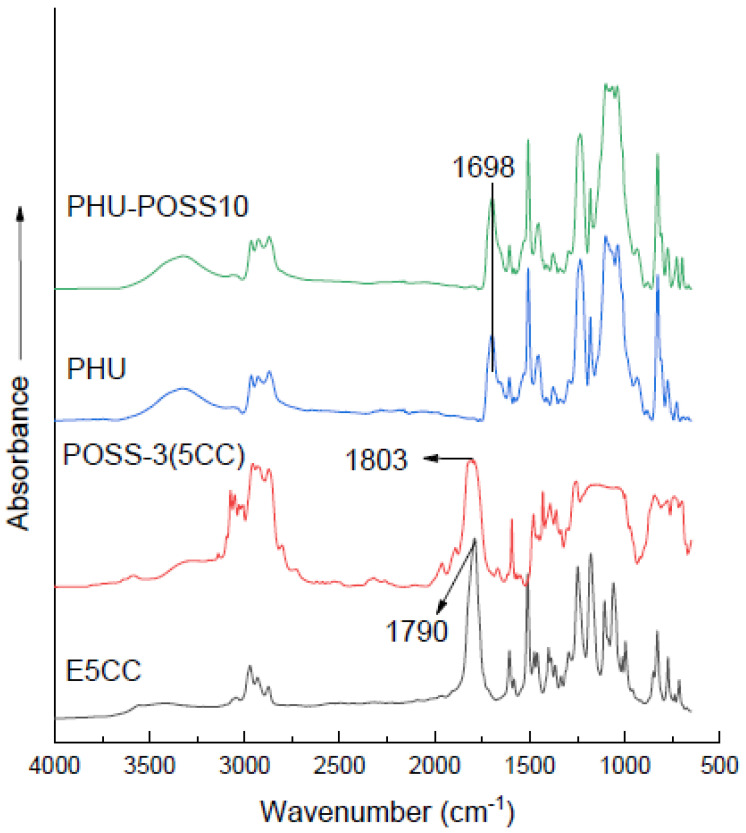
FTIR spectra of DE5CC, POSS-3(5CC), PHU, and PHU-POSS10 nanocomposite.

The novel POSS macromer, POSS-3(5CC) was introduced into PHU to obtain the organic-inorganic composites. The control PHU network was obtained via the crosslinking reaction of a difunctional five-member cyclic carbonate which was synthesized from diglycidyl ether of bisphenol A (denoted DE5CC) and carbon dioxide (CO_2_) with a trifunctional polyetheramine (i.e., Jeffamine T-403). To ensure sufficient reaction of DE5CC, POSS-3(5CC) with Jeffamine T-403, the crosslinking reactions were started from the mixtures of DE5CC, POSS-3(5CC), Jeffamine T-403 with tetrahydrofuran. The reaction was carried out under the refluxing condition of tetrahydrofuran. Notably, the system was gradually gelled as the reaction proceeded. After the removal of solvent, the gelled products can be further processed (this will be addressed later). The PHU-POSS composites were subjected to Fourier transform infrared (FTIR) spectroscopy as shown in Figure 3. It is seen that DE5CC displayed the infrared absorption band at 1790 cm^−1^, which was attributable to the stretching vibration of the carbonyl group of five-membered cyclic carbonate. In the case of POSS-3(5CC), the stretching vibration band of the carbonyl group slightly shifted to 1803 cm^−1^. After the crosslinking reaction of DE5CC or/and POSS-3(5CC) with T403, notably, the carbonyl stretching bands fully disappeared; concurrently there appeared the new bands at 1698 cm^−1^, assignable to the stretching vibration of carbonyl groups in urethane structural units. The FTIR spectroscopy indicates that PHU and/or PHU-POSS networks were successfully obtained.

**Figure 4:**
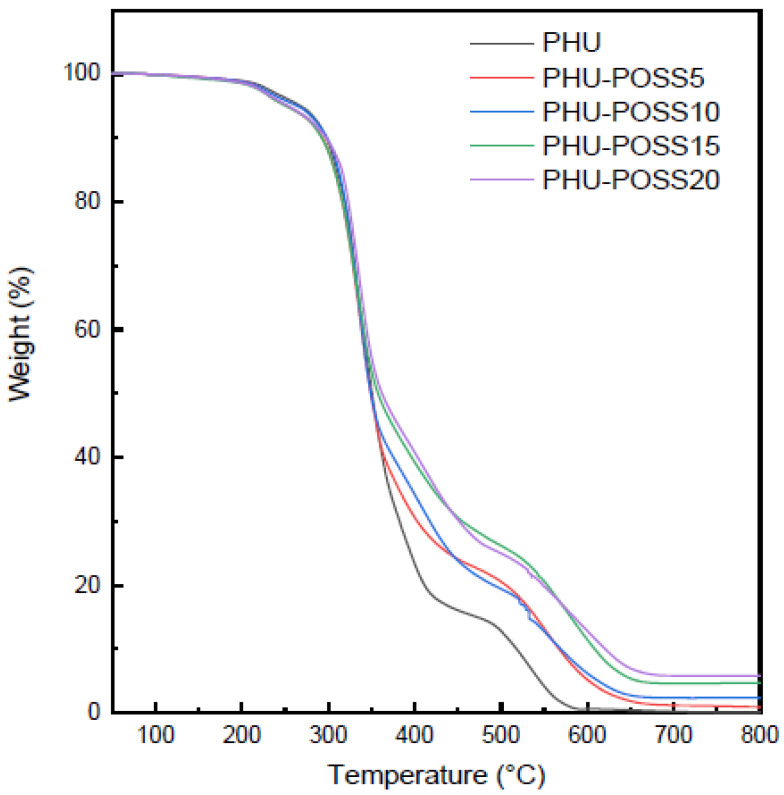
TGA curves of PHU and PHU-POSS nanocomposites.

The control PHU and organic-inorganic composites were subjected to thermal gravimetric analysis (TGA). The TGA measurements were carried out with air atmosphere. In all cases, the two-step curves of degradations were displayed (See Figure 4). The first ones appeared in the range of 300~500 °C, which are assignable to the thermo-oxidative degradation of organic components. The second ones were detected at 600~700 °C, assignable to the final decomposition of char. Notably, the PHU-POSS nanocomposites had almost the same initial degradation temperatures (*T*_d_’s), suggesting that the introduction of POSS cages did not significantly affect the thermal degradation of PHU networks. However, the composites displayed the retarded degradation or decomposition in the range of 600~700 °C. The higher the contents of POSS, the more pronounced the retardance of flame, indicating that the introduction of POSS significantly enhanced the thermal stability of PHU. The improved thermal stability was attributable to the following factors: (i) POSS itself had the higher thermal resistance than PHU; (ii) the introduction of POSS cages in the place of PHU segments restricted the release of gaseous products generated during thermal degradation. For control PHU, notably, the degradation, as well as decomposition, was undergone completion at 800 °C. For the PHU-POSS composites, however, there were still the residues of decomposition, the yield of which increased with increasing the contents of POSS. Assuming that the residues were only the inorganic component (i.e., silica, SiO_2_), which was rendered from the thermal degradation and oxidation of POSS cages, the yields of the degradation residues can be employed to calculate the contents of POSS cages in the composites with the following equation:

(1) $$W_{POSS(wt\%)}=\frac{M_{POSS}}{10\times M_{SiO_{2}}}\times W_{SiO_{2}(wt\%)}$$

where *W_POSS(wt%)_* is the POSS content in the nanocomposites, *M_POSS_* is the molecular weight of POSS, *M_SiO*_2_*_* is the molecular weight of SiO_2_, and *W_SiO*_2_*_* is the residue yield at 800 °C. The calculated POSS contents were 3.2, 6.6, 12.1, and 15.3 wt%, respectively, which were slightly lower than the theoretical values.

**Figure 5:**
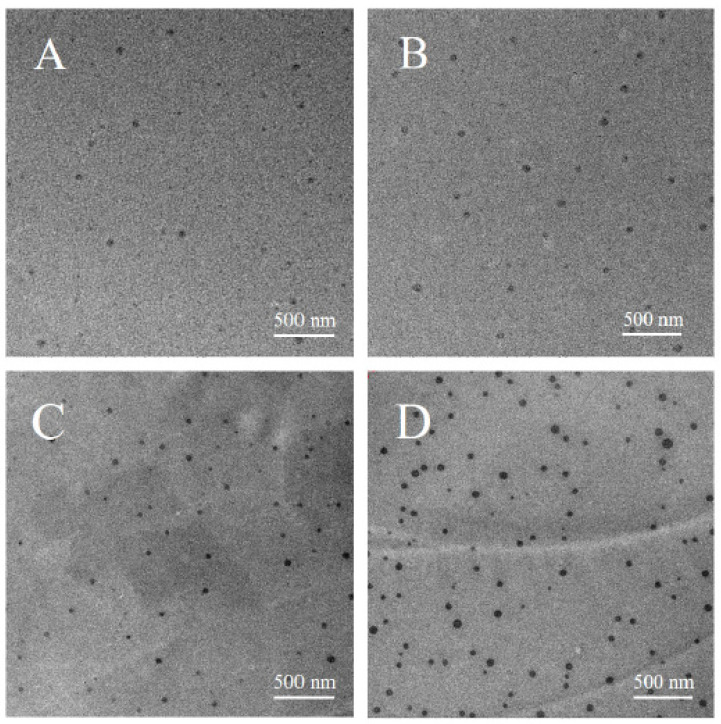
TEM images of: (**A**) PHU-POSS5; (**B**) PHU-POSS10; (**C**) PHU-POSS15 and (**D**) PHU-POSS20.

Transmission electron microscopy (TEM) was performed to investigate the morphologies of the PHU-POSS composites; the TEM images are shown in Figure 5. In all the cases, the heterogamous morphologies were exhibited. In terms of the difference in electron density between POSS and PHU, the dark domains are attributed to POSS while the shallow to PHU. The TEM results showed that the POSS cages existed in the organic-inorganic composites in the form of POSS microdomains. In the other words, the POSS cages were aggregated into the spherical nanoobjects with the size of 20~40 nm in diameter. In terms of the size of a single POSS cage, it is proposed that the POSS microdomains were the aggregates of tens of POSS cages. It is seen that with increasing the contents of POSS, the number of POSS microdomains increased whereas the sizes remained almost unchanged. The POSS microdomains were created via POSS-POSS interactions, i.e., the POSS component was immiscible with PHU. The immiscibility could give rise to the occurrence of macroscopic phase separation. However, there were chemical linkages between POSS cages with PHU chains and thus the tendency of macroscopic phase separation was suppressed. A similar formation of POSS microdomains has been found in the organic-inorganic PHU hybrids in which the POSS macromer was completely condensed cages [52]. Notably, the POSS microdomains were still rendered although the POSS macromer used in this work is an incompletely condensed POSS cage.

**Figure 6:**
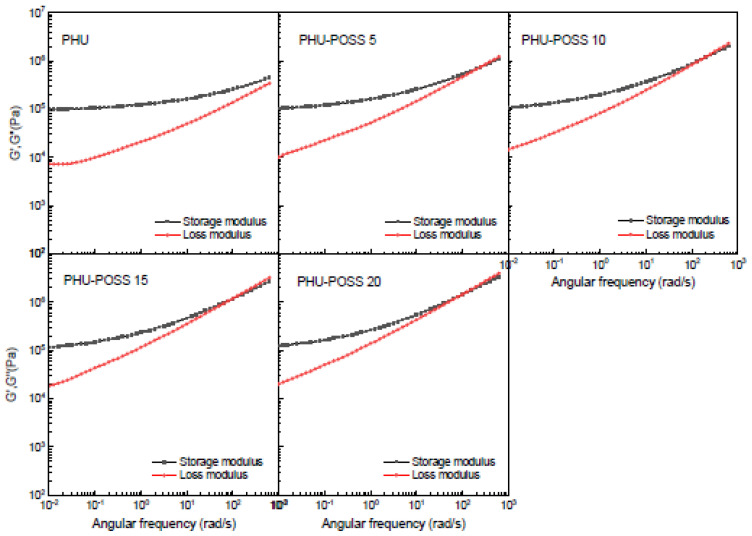
Rheological data of PHU and PHU-POSS nanocomposites with an angular frequency sweep at 80 °C.

**Figure 7:**
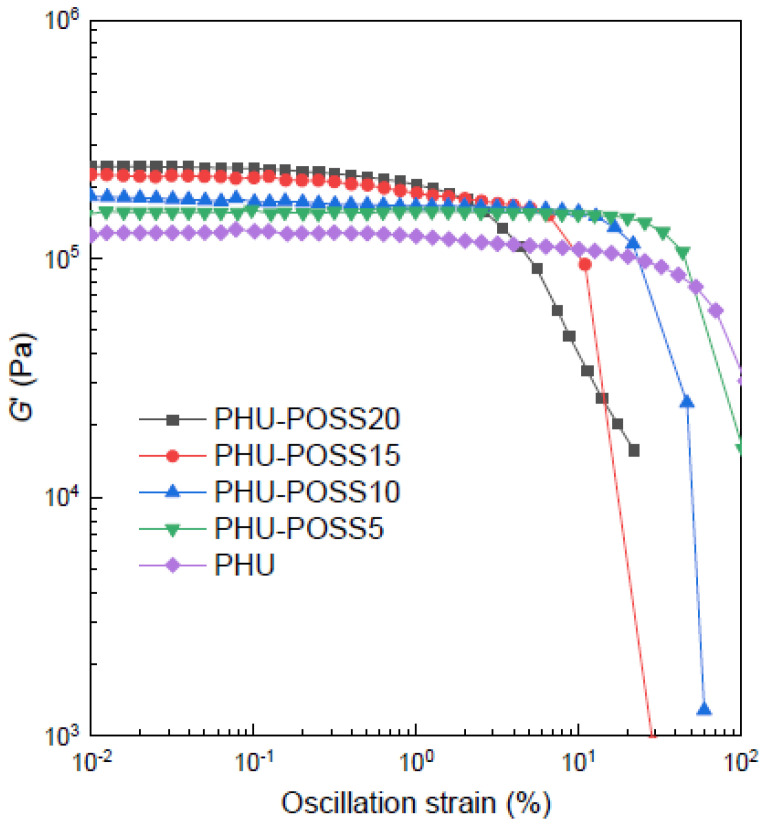
Rheological data of PHU and PHU-POSS nanocomposites with a strain sweep.

The POSS-POSS interactions were further investigated by means of rheological analysis. Figure 6 shows the shear frequency sweep data for the PHU and nanocomposites at 80 °C. In all the cases, the dynamic storage and loss modulus (i.e., *G*′ and *G*″) was frequency-dependent. The values of *G*′ were higher than that of *G*″, indicating that the samples displayed the properties of elastic solids due to the generation of crosslinked networks. Notably, the values of *G*′ increased with increasing the contents of POSS. The increased *G*’ values are interpreted on the basis of the following aspects. On the one hand, the POSS microdomains have exerted the nanoreinforcement on the PHU matrix, which causes the enhancement of modulus. On the other hand, the POSS-POSS interactions with the POSS microdomains constituted the additional crosslinking, which resulted in the increase in modulus. To demonstrate the nature of physical interactions caused by the POSS microdomains, the rheological measurements with strain amplitude sweep were carried out and the data were presented in Figure 7. For the PHU, the linear viscoelastic region was measured with a strain as high as 50%. For all the nanocomposites, the linear viscoelastic regions were significantly narrowed although the dynamic storage moduli increased with increasing the content of POSS. The linear viscoelastic region decreased with increasing the contents of POSS. For PHU-POSS20, the linear viscoelastic region was diminished to 2.3%. The higher the contents of POSS, the lower the yield strains. It is proposed that the decrease in the linear viscoelastic region resulted from the re-organization of POSS microdomains under the increased strains. The introduction of POSS microdomains has exerted a significant impact on the thermal and mechanical properties.

### 3.3. Thermal and Mechanical Properties

**Figure 8:**
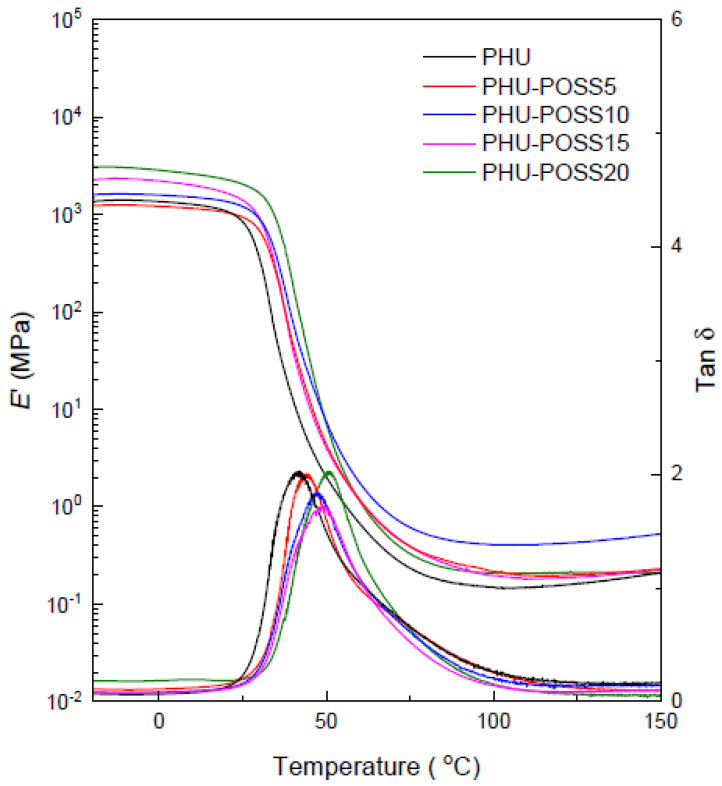
DMTA curves of PHU and PHU-POSS nanocomposites.

The thermal properties of PHU and PHU-POSS nanocomposites were studied by dynamic mechanical thermal analysis (DMTA). Figure 8 shows the storage modulus and tan δ curves of the samples. For PHU, one single major transition was detected at 41.2 °C, assignable to the glass transition of the crosslinked polymer. After the introduction of POSS, notably, the *T*_g_’s were enhanced; the *T*_g_’s increased with the increasing content of POSS. For the PHU-POSS20 sample, the *T*_g_ was increased to 51.1 °C. The increased *T*_g_’s are associated with the following two aspects. On the one hand, the increase in *T*_g_’s are ascribed to the nanoreinforcement of POSS microdomains on PHU matrices. The nanoreinforcement of POSS microdomains restricted the segmental motion responsible for glass transition. On the other hand, the increase in *T*_g_’s is accounted for the generation of additional crosslinking with the POSS microdomains as the crosslinking sites. The additional crosslinking also constituted the restriction on the segmental motion of PHU. It should be noted that only the single *T*_g_’s were detected although the TEM results showed that the PHU-POSS nanocomposites were heterogeneous. A possible explanation is that the *T*_g_’s of POSS microdomains were too high to be tested. The nanoreinforcement of POSS microdomains and the additional crosslinking caused with the POSS microdomains were also evidenced by the fact that the storage moduli (*E*’) in the glassy and rubbery states were all higher than that of PHU.

**Figure 9:**
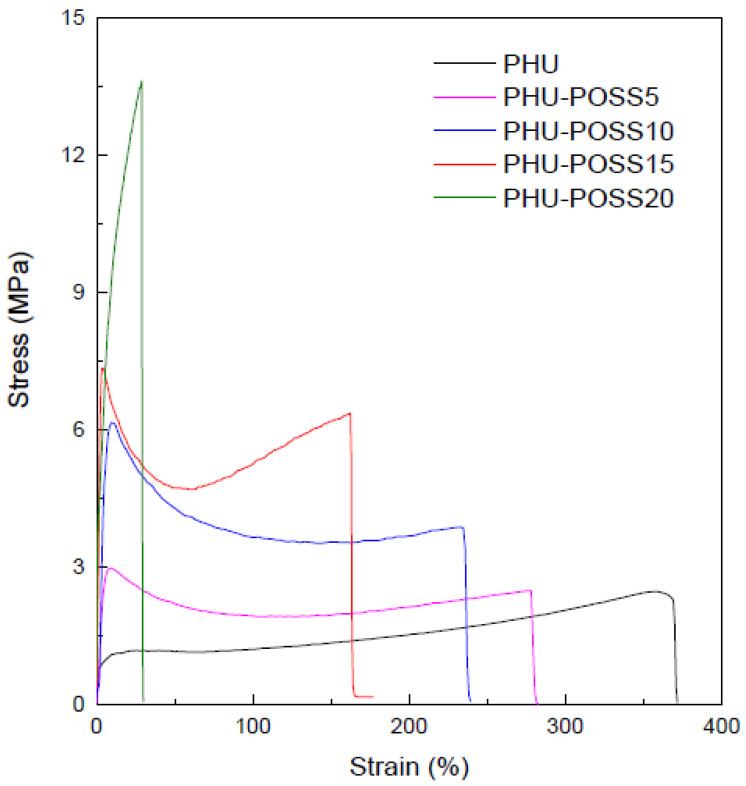
Stress-strain curves of PHU and PHU-POSS nanocomposites.

The impact of POSS microdomains on the mechanical properties of the PHU-POSS nanocomposites is investigated with tensile tests; the strain-stress curves are shown in Figure 9. For PHU, an unobvious yield phenomenon was observed and the elongation at break as high as *ε*_b_ = 370% was exhibited. This observation is attributable to the fact that the PHU had the glass transition temperature (*T*_g, DMTA_ = 41 °C) quite close to room temperature (i.e., testing temperature). Under this circumstance, there was intense segmental motion, which hindered the occurrence of forced high-elastic deformation. Upon introducing POSS, the segmental motions were restricted. Consequently, forced high-elastic deformation (viz. yield phenomena) occurred; the yield behavior was exhibited for PHU-POSS5, PHU-POSS10, and PHU-POSS15. Notably, the stress at the yield point increased with increasing the contents of POSS. For PHU-POSS20, a brittle fracture occurred due possible to the too strong nanoreinforcement of POSS microdomains. Notably, Young’s modulus and tensile strength increased with increasing the contents of POSS which was a result of the nanoreinforcement of POSS microdomains (See Table 1). In addition, upon introducing POSS, the elongations at break were significantly decreased. The decreased elongation at break is ascribed to the formation of the microphase-separated morphologies.

### 3.4. Reprocessing Properties

**Figure 10:**
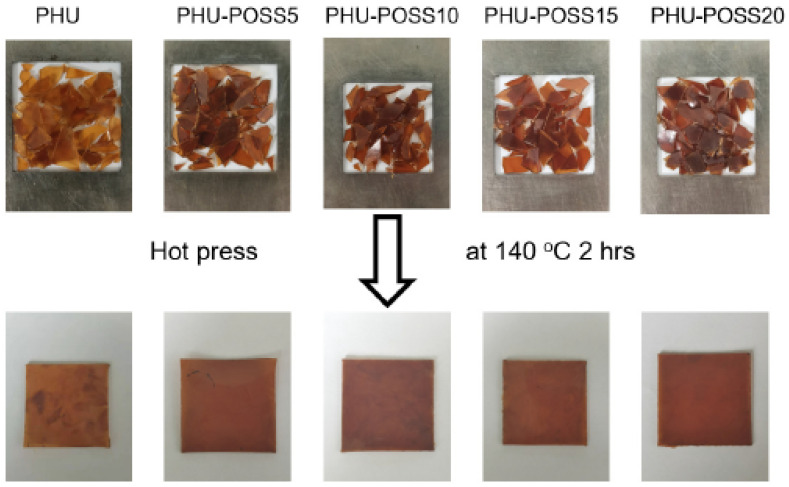
Photographs of PHU and PHU-POSS nanocomposites for reprocessing tests.

**Figure 11:**
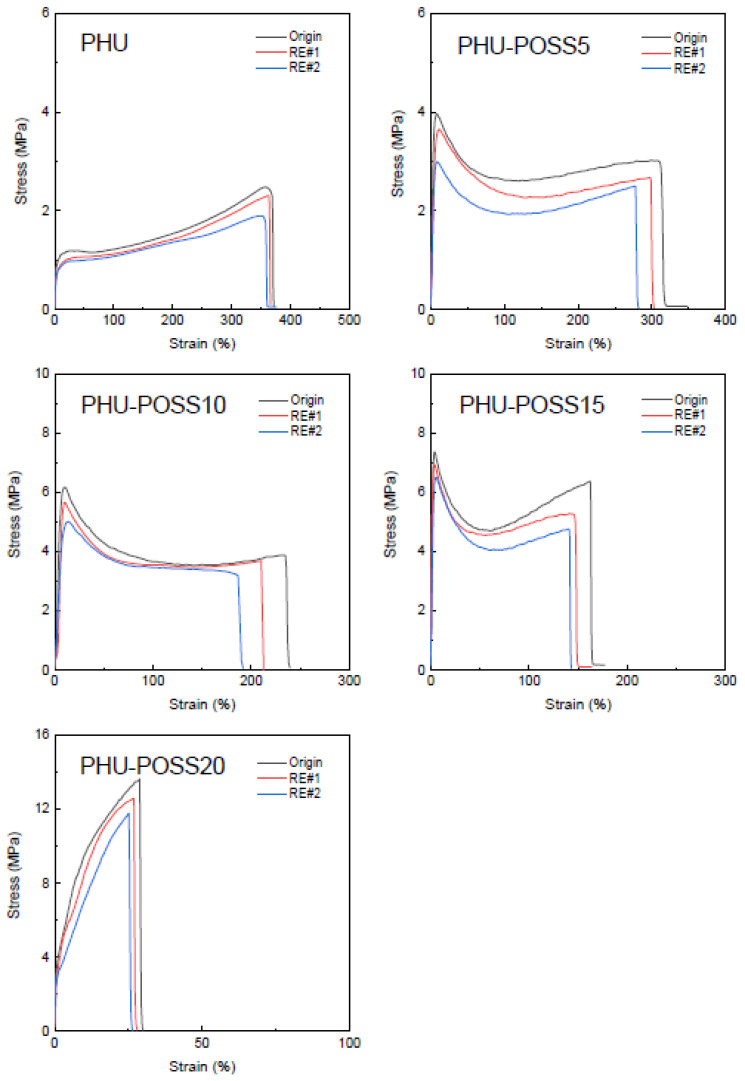
Stress-strain curves of PHU and PHU-POSS nanocomposites for reprocessing tests.

It is known that PHU thermosets can display reprocessing properties at elevated temperatures [35,36]. For the PHU thermosets synthesized from six-membered cyclic carbonates and multifunctional amines, the reprocessing properties are associated with transcarbamoylation of side hydroxyl with carbamate groups [35]. For those resulting from five-membered cyclic carbonates, Torkelson et al. [36] demonstrated that reprocessing properties were additionally attributable to reversible cyclic carbonate aminolysis. In the present work, we investigated the reprocessing properties of PHU and PHU-POSS nanocomposites. Toward this end, all the samples were cut into small pieces, which were then pressed under 10 MPa at 140 °C. Notably, all the small pieces can be mended into monolithic bodies without discernable defects (Figure 10). To examine the mending degree of the specimens, the tensile tests of the reprocessed samples were carried out. Compared with the original samples, the reprocessed samples displayed slightly decreased tensile strength, Young’s moduli, and elongations at break (Figure 11). Herein, we calculated the recovery of mechanical property (*F*) by comparing the fracture energy of the samples, which are defined as the integral area below the stress-strain curves [37]. For the first and second reprocessing of control PHU, the *F* values were calculated to be 90.6% and 80.5%, respectively. Upon introducing POSS, notably, the *F* values were slightly reduced. For the PH-POSS20 nanocomposite, the *F* values were obtained to be 84.5 and 67.9%, respectively after the sample was processed twice and thrice. This observation can be interpreted on the basis of the following two aspects. First, the introduction of POSS decreased the density of dynamic exchange bonds, leading to the decrease of the recovery of mechanical properties. Second, the formation of POSS microdomains restricted the segmental motion of PHUs, as a result, the activation energy of the dynamic exchange reaction increased.

**Figure 12:**
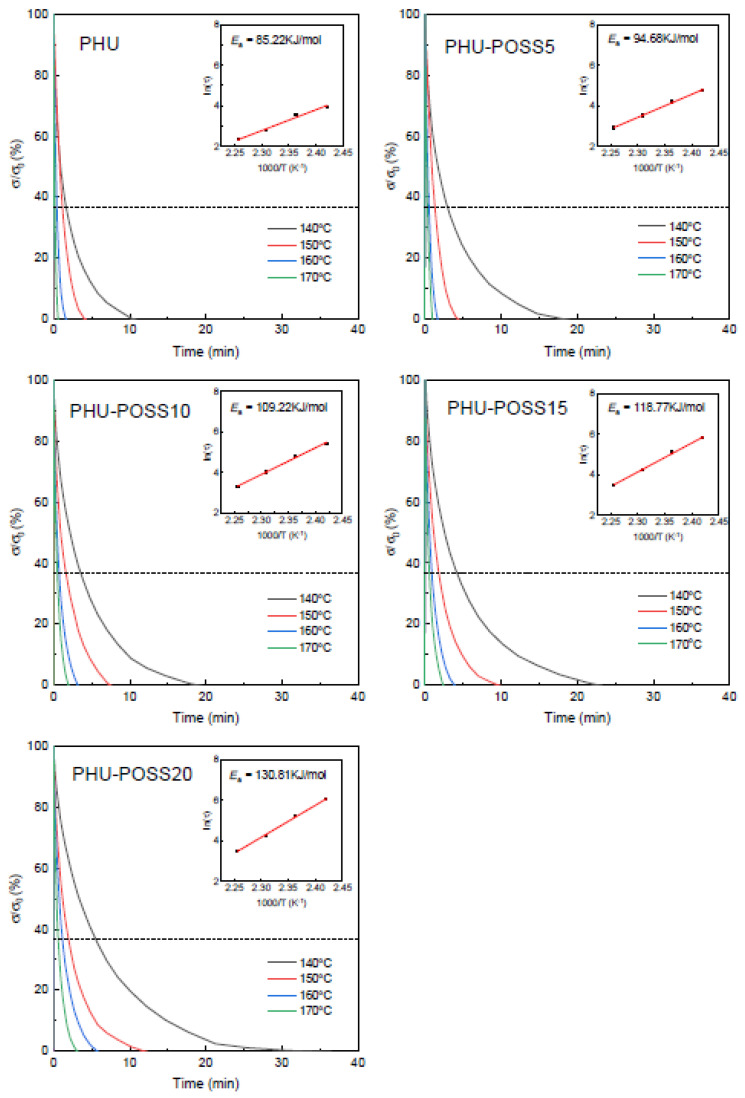
Stress relaxation curves of PHU and PHU-POSS nanocomposites at various temperatures.

The influence of the POSS on the reprocessing properties was further investigated by the use of stress relaxation measurements. The stress relaxation curves at different temperatures were shown in Figure 12. At the measured temperature, the stress of all the samples can be relaxed to 0, indicating that the networks of nanocomposites can indeed be reprocessed. Notably, PHU-POSS nanocomposites displayed slower stress relaxation than PHU. The decelerated stress relaxation was determined by two factors. On the one hand, the introduction of POSS gave rise to the increase in overall crosslinking density since the POSS microdomains constituted the additional crosslinking. On the other hand, the POSS microdomains restricted the segmental motion of the PHU matrix, delaying the rearrangement of the network. According to the stress relaxation curves, the apparent activation energy (*E*_a_) for stress relaxation can be calculated by the Arrhenius equation [35,38]:

(2) $$\tau =Ae^{\left(-E_{a}/RT\right)}$$

where *τ* is stress relaxation time, *A* is the pre-exponential factor, and *R* is gas constant. The activation energy of PHU was *E*_a_ = 85.22 kJ/mol. Notably, the PHU-POSS nanocomposites displayed the enhanced *E*_a_’s values. The higher the POSS contents, the higher the *E*_a_’s values. While the POSS content was 20 wt%, the value of *E*_a_ was increased to 130.81 kJ/mol. The increased *E*_a_ values indicate that the PHU matrices in the nanocomposites were significantly reinforced! Nonetheless, the organic-inorganic nanocomposites of PHU with POSS still displayed reprocessing properties.

## 4. Conclusions

In summary, a novel trifunctional POSS five-membered cyclic carbonate [POSS-3(5CC)] was successfully synthesized. The POSS macromer was successfully introduced into the PHU networks and the organic-inorganic nanocomposites of PHU with POSS were successfully obtained. The nanocomposites had microphase-separated morphologies and the POSS cages were self-organized into the spherical POSS microdomains via POSS-POSS interactions with the size of 20~40 nm in diameter. Compared to control PHU, the PHU-POSS nanocomposites displayed the improved Young’s modulus and tensile strength. Notably, the PHU-POSS nanocomposites still preserved reprocessing properties although the recovery of the mechanical properties was slightly decreased with the introduction of POSS.

## Figures and Tables

**Table 1:** Thermal and mechanical properties of PHU and PHU-POSS nanocomposites.

Samples	Glass Transition Temperature *T*_g_ *^a^* (°C)	Degradation Temperature *T*_d_ *^b^* (°C)	Tensile Strength (MPa)	Elongation at Break (%)	Young’s Modulus (MPa)
PHU	41.2	299.5	2.5 ± 0.2	369 ± 31	10.7 ± 0.9
PHU-POSS5	44.1	298.6	2.8 ± 0.2	277 ± 27	29.6 ± 1.4
PHU-POSS10	46.9	299.2	3.9 ± 0.3	234 ± 16	47.2 ± 2.9
PHU-POSS15	48.7	298.7	6.4 ± 0.3	161 ± 18	59.2 ± 3.6
PHU-POSS20	51.1	299.6	27.7 ± 0.7	30 ± 7	89.9 ± 4.5

*a*: glass transition temperature measured by DMTA. *b*: degradation temperature at 10wt% of weight loss measured by TG.

## Data Availability

Not applicable.
